# Parastomal herniation of the entire gallbladder and postoperative complications from bile leakage via a subvesical bile duct

**DOI:** 10.1093/jscr/rjag575

**Published:** 2026-07-13

**Authors:** Emil L Villumsen, Taatsiannguaq I Olsen, Ester B J Nielsen, Magnus Ploug

**Affiliations:** Department of Gastrointestinal Surgery, Esbjerg Hospital, University Hospital of Southern Denmark, 6700 Esbjerg, Denmark; Department of Clinical Medicine - Comparative Medicine Lab, Aarhus University, 8200 Aarhus N, Denmark; Department of Gastrointestinal Surgery, Esbjerg Hospital, University Hospital of Southern Denmark, 6700 Esbjerg, Denmark; Department of Gastrointestinal Surgery, Esbjerg Hospital, University Hospital of Southern Denmark, 6700 Esbjerg, Denmark; Department of Gastrointestinal Surgery, Esbjerg Hospital, University Hospital of Southern Denmark, 6700 Esbjerg, Denmark; Department of Regional Health Research, University of Southern Denmark, 5230 Odense, Denmark

**Keywords:** gallbladder, parastomal herniation, subvesical bile duct, gallbladder herniation

## Abstract

Parastomal herniation (PSH) is a common complication after establishing an enterotomy. The hernia typically contains omentum, small bowel, colon, and rarely other viscera. We present the case of an 83-year-old woman with PSH of the entire gallbladder containing gallstones with acute cholecystitis and necrosis due to torsion of the gallbladder neck. Surgery was complicated by fascial dehiscence with reactive small bowel obstruction caused by bile leakage from a subvesical bile duct. The case illustrates the high level of complexity related to the surgical treatment of organ herniation and raises awareness that such surgery poses significant risks of postoperative complications.

## Introduction

A parastomal herniation (PSH) is a common occurrence after establishing an enterostomy with a rate of up to 58% depending on stoma type and follow-up duration [1]. PSH is a herniation between the stoma and the abdominal fascial aperture, typically involving small-bowel loops or part of the greater omentum [2]. The increase in PSH rates over longer follow-up periods is thought to result from gradual weakening and lengthening of the connective tissue over time [3]. Other risk factors include increased abdominal pressure and corticosteroid treatment for chronic obstructive pulmonary disease (COPD) [3]. A very rare subset of PSH involves herniation of the gallbladder. This subtype predominantly affects elderly females [4]. Asymptomatic patients may qualify for conservative treatment, but cases usually warrant surgical treatment due to serious conditions such as acute cholecystitis, gallbladder torsion, incarceration of the gallbladder, and even perforation of the gallbladder with peritonitis [4]. When removing the gallbladder care must be taken to avoid damaging subvesical bile ducts. Subvesical bile ducts are small (mean diameter 2 mm) bile ducts directly between the gallbladder and the liver, prevalent in 4% of the population [5]. When these ducts are not perioperatively acknowledged and ligated, they can cause postoperative cholascos and peritonitis.

## Case report

We present the case of an 83-year-old woman with COPD, atrial fibrillation, and morbid obesity, who had previously (2006) received a total colectomy with end-ileostomy as treatment for ulcerative colitis. Due to subsequent abdominal herniation, a combined surgical repair of a midline hernia and PSH was performed in 2017. Yet, the PSH recurred and the patient was re-evaluated in 2022, 2023 and 2024, where a conservative/non-surgical approach was taken due to multiple co-morbidities, most notably morbid obesity. The patient was eventually scheduled for PSH repair in June of 2025 after weight loss, but because of recently diagnosed disseminated cervical cancer, surgery was cancelled. The patient had previously, in both 2023 and 2024, presented with small-bowel obstruction (SBO) related to the stoma, which was resolved with conservative treatment. Gallstones were acknowledged as early as 2017.

In the fall of 2025, the patient presented in the emergency department with 4 days of progressively worsening pain in her upper right abdomen. Clinical evaluation confirmed a large PSH and a computed tomography (CT) scan showed PSH of small-bowel, part of the right liver lobe along with the entire enlarged gallbladder containing gallstones, shown in Fig. 1.

**Figure 1:**
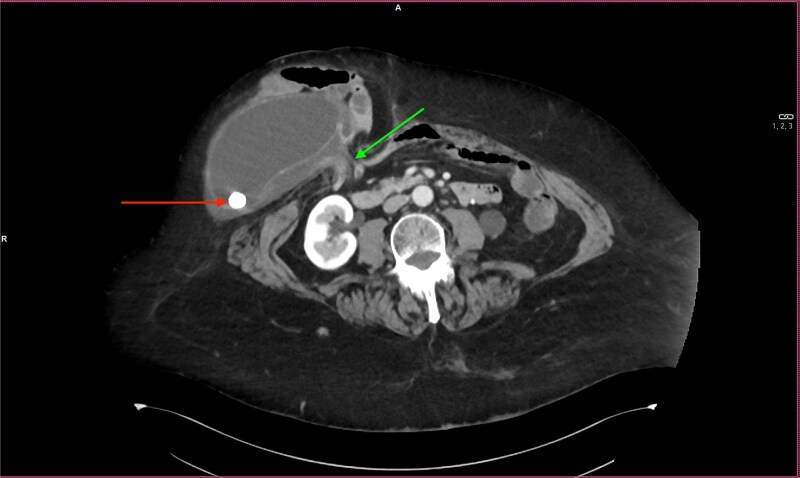
Transverse CT slice showing the entire enlarged gallbladder in a parastomal hernia. Leftmost arrow: gallstone. Rightmost arrow: fascial aperture through which the abdominal contents herniate.

Cholecystitis was suspected on CT due to reactive edema surrounding the gallbladder and elongated gallbladder mesentery, increasing the risk of torsion, shown in Fig. 2. Due to stable vitals and inconspicuous bloodwork, a conservative approach was attempted while her apixaban was paused to allow for surgery. Three days later, due to failing clinical improvement and rising inflammatory markers [leucocytes and C-reactive protein (CRP)], surgery was scheduled.

**Figure 2:**
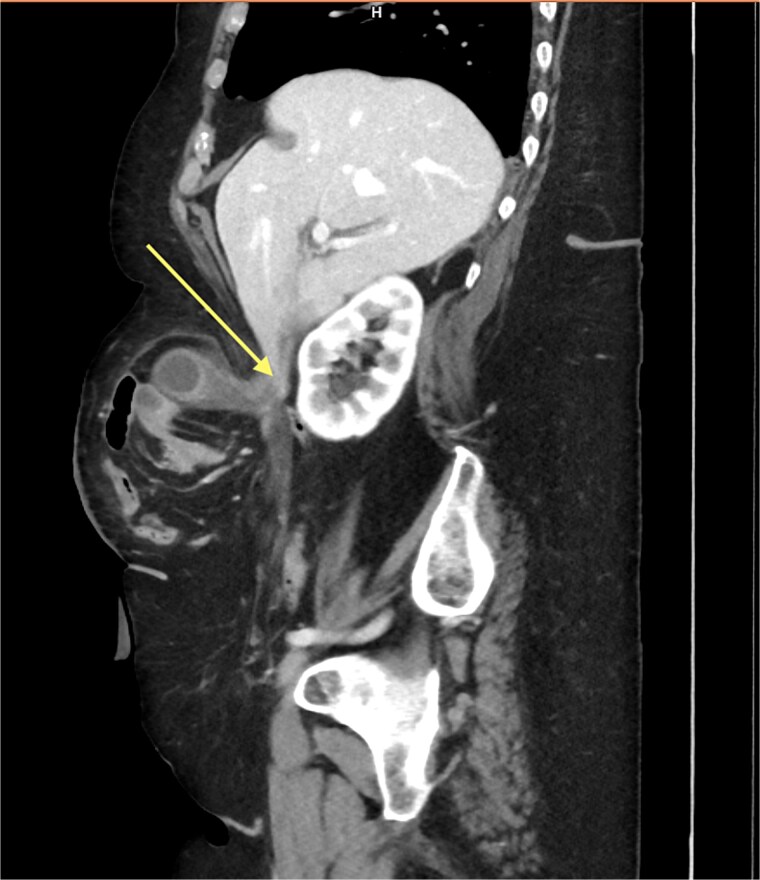
Sagittal CT slice showing the herniation through the abdominal wall. Arrow: elongated mesentery of the gallbladder.

A laparoscopic approach was attempted, but severe adhesions in the hernia warranted conversion to laparotomy. The gallbladder, torqued and exhibiting necrotic areas, was removed after the fascial aperture surrounding the stoma had been slightly expanded. Adhesions between the small bowel and the abdominal wall, hernia sac and between segments of small bowel were redeemed, and the small bowel was repositioned. The PSH aperture was reduced with four sutures of Ethibond 0.

Six days following index surgery, suspicion of fascial dehiscence and intra-abdominal bleeding arose due to a seeping bleeding from the caudal part of the laparotomy incision. A renewed CT scan revealed suspicion of subcutaneous bleeding, fascial dehiscence, and free intrabdominal fluid. Consequently, the patient was re-operated revealing adhesions and SBO in relation to a caudal fascial dehiscence. Peritonitis in the upper right quadrant of the abdomen was identified due to bile leakage caused by a subvesical bile duct not identified during the index surgery. The bile duct was electrocoagulated, and hemostatic gauze was applied. The abdomen was closed using a vacuum assisted closure system with a second look scheduled 2 days later. During the second look operation, the gallbladder bed was again found to produce bile from the previously identified subvesical bile duct. The duct was definitively closed using Nylon 4.0 suture, and an 18 Fr drain was placed at the site due to concerns about postoperative bile leakage. The patient had 1 day in the ICU and was discharged 12 days after the final operation with no further surgical complications.

## Discussion

Herniation of the gallbladder into a PSH is exceedingly rare with only 19 previously documented cases in the literature [2, 4, 6]. Cases have pertained primarily to elderly female individuals (mean age 74.9 years; 85% female) presumably due to the natural weakening of connective tissue and elongation of the gallbladder mesentery [2, 4, 7, 8]. Most have been in ileal conduits or ileostomies (80%). Operative management was undertaken in 14 of 20 reported cases (70%). Among these, open gallbladder surgery predominated, with open cholecystectomy or cholecystostomy performed in nine cases (64.3% of operative cases; 45% of overall management strategies) [2, 6, 8].

In this case the patient had an open cholecystectomy with sutured PSH repair and no mesh placement. The aim of the cholecystectomy was to remove her torqued, necrotic gallbladder, whereas PSH repair was seen as a secondary measure and mesh placement in a potentially contaminated field was unwanted. The postoperative course was complicated by leakage from a subvesical bile duct leakage, and subsequently by failure to secure initial closure with electrocoagulation. Consequently, the patient experienced a total of three operations. Definitive intraoperative management of a producing, discernible subvesical bile duct should preferably be clips or ligation. In cases with limited field of view identification and targeted, closure can be difficult. In cases where complete closure cannot be obtained, an appropriate step-up could be sphincterotomy of the sphincter of Oddi [9]. Surgical management of PSH containing gallbladder should be subject to individual risk assessments taking co-morbidities and symptoms into account because of its rarity.

## References

[1] Antoniou SA, Agresta F, Garcia Alamino JM et al. European Hernia Society guidelines on prevention and treatment of parastomal hernias. Hernia 2018;22:183–98. 10.1007/s10029-017-1697-5 29134456

[2] Pinnock N, Vashi A, Marsh JW et al. Spontaneous resolution of parastomal gallbladder herniation after attempted surgical intervention: a case report and review of the literature. Cureus 2023;15:e37355. 10.7759/cureus.37355 37182048 PMC10170294

[3] Moeckli B, Limani P, Clavien PA et al. Parastomal gallbladder herniation: a case report and review of the literature. Int J Surg Case Rep 2020;73:338–41. 10.1016/j.ijscr.2020.07.002 32739522 PMC7397696

[4] Olusola S, Jarman T, Parmar C et al. Parastomal gallbladder herniations: a systematic review. Cureus 2024;16:e71379. 10.7759/cureus.71379 39539891 PMC11558021

[5] Schnelldorfer T, Sarr MG, Adams DB. What is the duct of Luschka?—a systematic review. J Gastrointest Surg 2012;16:656–62. 10.1007/s11605-011-1802-5 22215244

[6] Macanovic P, James-Knights Z, Saidy A et al. A Stoma's stowaway: a rare case of parastomal herniation of the gallbladder. Cureus 2025;17:e97642. 10.7759/cureus.97642 41450411 PMC12729440

[7] Bakshi C, Ruff S, Caliendo F et al. Acute cholecystitis in a parastomal hernia causing a small bowel obstruction. J Surg Case Rep 2017;2017:rjx235. 10.1093/jscr/rjx235 29423157 PMC5798022

[8] Bakmiwewa SM, Vaska A, Idrees M et al. Gallbladder in the wrong neighbourhood: tackling para-urostomal herniated cholecystitis with open cholecystostomy. Cureus 2025;17:e79543. 10.7759/cureus.79543 40144441 PMC11938171

[9] Carannante F, Mazzotta E, Miacci V et al. Identification and management of subvesical bile duct leakage after laparoscopic cholecystectomy: a systematic review. Asian J Surg 2023;46:4161–8. 10.1016/j.asjsur.2023.04.031 37127504

